# Temporal cytokine storm dynamics in dengue infection predicts severity

**DOI:** 10.1016/j.virusres.2023.199306

**Published:** 2024-01-06

**Authors:** Puneet Bhatt, Muralidhar Varma, Vikas Sood, Anoop Ambikan, Anup Jayaram, Naren Babu, Soham Gupta, Chiranjay Mukhopadhyay, Ujjwal Neogi

**Affiliations:** a Manipal Institute of Virology, Manipal Academy of Higher Education, Manipal, Karnataka, India; b Dept of Infectious Diseases, Kasturba Medical College, Manipal, Karnataka, India; c The Systems Virology Lab, Division of Clinical Microbiology, Department of Laboratory Medicine, Karolinska Institutet, Stockholm, Sweden; d Department of Biochemistry, School of Chemical and Life Sciences, Jamia Hamdard, Delhi, India; e Center for Emerging and Tropical Diseases, Manipal Academy of Higher Education, Manipal, Karnataka, India

**Keywords:** Cytokine storm, Dengue severity, Temporal Analysis

## Abstract

The immunopathogenesis of dengue severity is convoluted. The primary objective of the research was to examine the dynamics of cytokine storm and its correlation with disease development in individuals affected by DENV infection. Additionally, the study aimed to discover potential biomarkers that could indicate severe dengue infection and determine the most suitable timeframe for predicting the severity of these biomarkers during the acute stage of dengue infections. We conducted a temporal analysis of the daily viral load and cytokine levels in 60 hospitalized dengue patients until discharge. Our findings reveal a distinct cytokine profile (elevated IL-8, IL-10, IL-6, GM-CSF, MCP-1, IL-13, and IL-4 and decreased IL-12, MIP-1β) on the third day after symptom onset is predictive of severe dengue in secondary dengue infection. The imbalanced cytokine signature may inform clinical decision-making in treating severe dengue infections.

## Highlights

• Temporal analysis of the daily viral load and cytokine levels in hospitalized dengue patients identified the pattern of cytokine dynamics.
• Elevated IL-8, IL-10, IL-6, GM-CSF, MCP-1, IL-13, and IL-4 and decreased IL-12, MIP-1β on the third day after symptom onset is predictive of severe dengue.
• The imbalanced cytokine signature may inform clinical decision-making in treating severe dengue infections.

Dengue virus (DENV) belongs to the Flaviviruses family and is a major global health concern. In 2023, 4.1 million suspected dengue cases were reported globally, of which 6710 were classified as severe, leading to 2049 reported deaths (Dengue-Global Situation, 21 December 2023, https://www.who.int/emergencies/emergency-events/item/2023-DON498). The clinical manifestations of Dengue can be divided into three phases: febrile, defervescence, and recovery or convalescent phase. The febrile phase, usually self-limiting, typically lasts 2–7 days and is characterized by sudden onset of high fever, headache, muscle and joint pain, and rash. Patients may also experience nausea, vomiting, and loss of appetite. The defervescence phase typically begins when the fever subsides, usually between days 3 and 7 of illness. Plasma leakage and hemorrhage leading to severe DENV infection are characteristics of a certain subset of patients (Chunhakan et al., 2015). The recovery or the convalescent phase of Dengue occurs in patients who do not progress to the critical phase. During this phase, patients typically experience a gradual resolution of symptoms over 2–5 days. However, patients may experience prolonged fatigue and weakness lasting several weeks.

The pathogenesis of severe Dengue is complex and poorly understood, but it is thought to involve an exaggerated immune response to the virus, leading to cytokine storms. Excessive release of pro-inflammatory cytokines may contribute to vascular permeability, thereby contributing to the severity of the disease (Patro et al., 2019). This increase in vascular permeability can cause plasma leakage and other complications such as shock, bleeding, and organ failure. Studies have shown that in addition to pro-inflammatory cytokines, there was an increase in anti-inflammatory cytokines such as interleukin-10 (IL-10) and transforming growth factor-beta (TGF-β) (Fiestas Solórzano et al., 2022; Tsai et al., 2013). Identifying pro- and anti-inflammatory cytokine since the onset of symptoms can predict dengue severity for clinical decision-making.

Several previous studies have investigated the cytokine response in Dengue patients. However, most studies involve stratification of the patients according to the Dengue severity and then measuring the cytokine levels crossectional (Imad et al., 2020). The only longitudinal cytokine profiles during febrile, defervescence, and convalescent stages grouped the patients into dengue fever (DF) and dengue hemorrhagic fever (DHF) (Kumar et al., 2012). Though highly valuable, this approach might not shed light on the kinetics of the disease progression as the duration of the phases of dengue virus infection differs significantly depending upon the host's immune status and is heterogeneous.

The study aimed to investigate the dynamics of cytokine storm and their relationship with disease pathogenesis in patients with DENV infection. We also aimed to identify potential biomarkers of severe dengue infection and determine the optimal time for predicting the severity biomarkers during acute dengue infections.

The blood samples were collected between May 2019 and April 2021 from Southern India, which is endemic for Dengue virus infection. The patients included were from the affiliated tertiary care hospital, various surveillance centers, and primary care hospitals in peripheral areas. Blood samples were collected immediately upon hospitalization and every day after that till the discharge of the patients and labeled based on the onset of their symptoms (days post symptoms). The inclusion criteria for the patients were hospitalized adults (age ≥18 years) with acute febrile illness (AFI), presented within three days of onset of fever with any other symptoms suggestive of dengue fever, like headache, body ache, retro-orbital pain, nausea/vomiting, petechiae/ecchymoses/purpura, bleeding gums, rash, abdominal pain, lethargy/restlessness. The exclusion criteria were not willing to provide informed consent and without a legally authorized representative available; Immuno-compromised cases, patients on chemotherapy or steroids or immunosuppressive drugs in the last one-year, post-transplant recipients; known cases of liver cirrhosis; co-infections (malaria, influenza, chikungunya, scrub typhus, leptospirosis, etc.). Laboratory confirmation of DENV infection was done by any one of the following:- ENV NS1 antigen ELISA (SD Bioline/Panbio), Dengue IgG capture ELISA (PanBio, USA), and IgM (MAC) ELISA (SD Bioline/Panbio) as per manufacturer's guideline. In patients with either dengue NS1 and/or IgM, positivity with high IgG levels indicates secondary dengue and can be detected as early as 3 days post onset of illness till 15 days. A positive result (>22 Panbio™ units) in Panbio™ Dengue IgG Capture ELISA was considered as active secondary infection (Santhosh Kumar et al., 2021). The viral load in the daily collected serum samples was estimated using real-time RT-PCR targeting the untranslated region (UTR) of DENV, as described previously (Gurukumar et al., 2009). Bio-Plex Pro-Human Cytokine 17-plex panel kit (Bio-Rad Laboratories Inc., USA) was used for the estimation of 17 cytokines (G-CSF, GM-CSF, IFN-γ, IL-1β, IL-2, IL-4, IL-5, IL-6, IL-7, IL-8, IL-10, IL-12 (p70), IL-13, IL-17, MCP-1, MIP-1β, TNF-α) in the human sera using Bio-Plex 200 system.

**Fig. 1:**
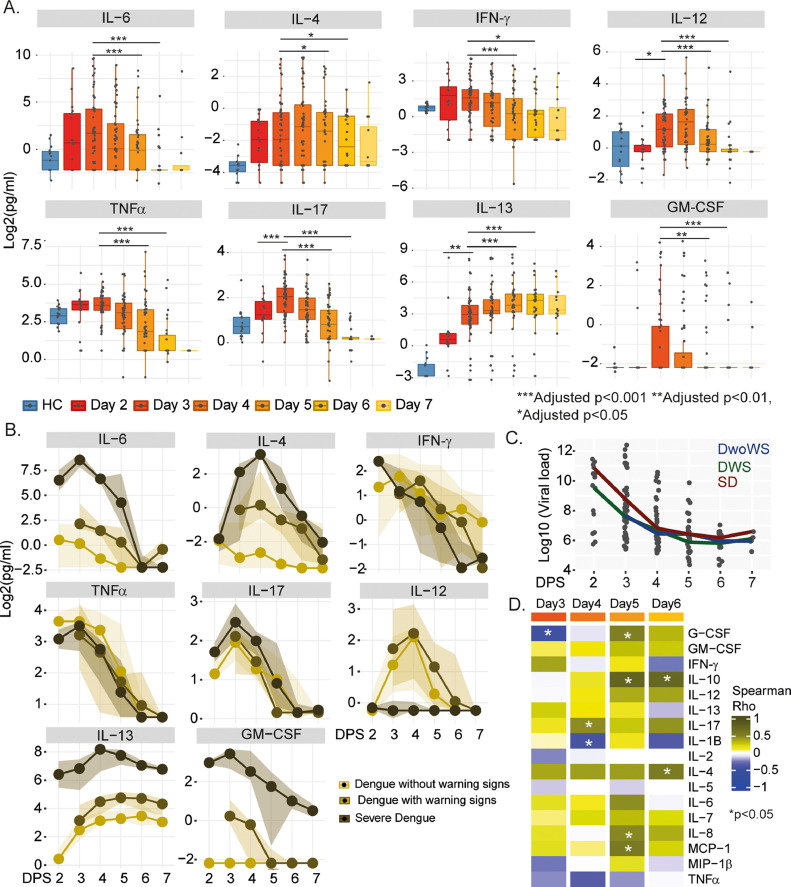
Temporal cytokine profiling in DENV patients. (A) Boxplot of the significant cytokines when a longitudinal analysis was performed between 3 and 5 DPS (*n* = 42 patients) or 3 to 6 DPS (*n* = 25 patients) where samples were available from all the patients. Asterisks represent significance in the Friedmann Test for repeated measure, and between 2 and 3 DPS (*n* = 17 patients) in Wilcoxon matched-pairs signed rank test (*p value<0.05, **<0.01, ***<0.001). (B) Line plot showing the dynamics of cytokine expression in three categories of dengue severity. The Y-axis represents the protein measurements [log2(pg/ml)], and the X-axis represents the number of days post symptoms. Points in the plot denote median measurement and the ribbon spans between the second and third quantiles. (C) Viral load dynamics over time. The line represents group specific median viral load levels at different days post symptoms. (D) Heatmap visualizes the correlation between cytokine expression and viral load measurement each day post symptoms. The color scale is relative to Spearman's correlation coefficient, and asterisks represent a significant correlation (p-value < 0.05).

A total of 66 patients were enrolled in the study. However, six patients were lost to follow-up, the remaining 60 patients were followed up till discharge from the hospital, and the severity of the disease was classified according to the WHO Dengue Case Classification 2009 . As per the WHO revised 2009 case classification, 44 patients were dengue without warning signs (DwoWS), 9 were dengue with warning signs (DWS), and 7 were severe dengue (SD) (Dengue: guidelines for diagnosis, treatment, prevention, and control, World Health organisation, 2009). All the severe and 5 of 9 DWS patients had secondary dengue infection. Additionally, samples from 10 healthy controls were also included in this study. Among the 17 cytokines tested, 12 were statistically significant (Friedmann Test, adjusted *p*<0.05) when we used three-time points 3–5 days post symptoms (DPS, *n* = 42 patients) or 11 cytokines when we used four-time points 3–6 DPS (*n* = 25 patients) (Supplementary Table 1) with eight proteins common in both the analysis (IL-6, IL-4, IFN-γ, IL-12, TNF-α, IL-17, IL-13, and GM-CSF) (Fig 1A). Only three proteins were significant at day two post symptoms (IL-12, IL-13, and IL-17, *n* = 17 patients). The severity-specific trajectories of individual cytokines were used for the temporal analysis and presented in Fig 1B. A temporal decrease in GM-CSF, IFN-γ, IL-17, IL-12, IL-6, and TNF-α levels was observed with the disease progression from 3 or 4 days onwards. We observed that the levels of IFN-γ were highly elevated on Day 2 of the illness and then progressively decreased with the progression of the disease, irrespective of severity. The results agreed to some extent with a previous study that showed that IL-4 and IFN-γ decreased as the disease progressed from the febrile phase to the defervescence phase (Kumar et al., 2012). The viral load also decreased from 3 DPS onwards, but no statistical significance was observed between the groups (Fig 1C). At the same time, there were a few non-specific correlations of the cytokines with the viral load and a significant positive correlation with IL-10 at 5 and 6 DPS, mainly the severe Dengue cases (Fig 1D).

**Fig. 2:**
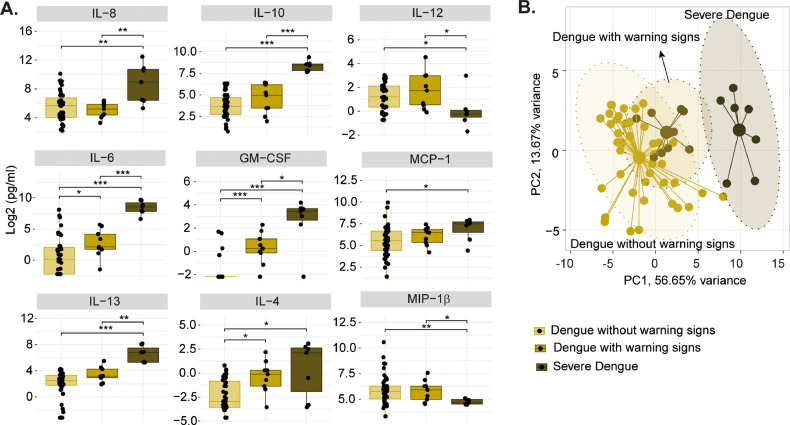
Biomarkers of the severe Dengue. (A) Boxplot showing the expression level of cytokines on the third-day post symptoms, which are found to be a predictive marker of severe dengue. Asterisks represent significance in the Mann-Whitney U test (*p value<0.05, **<0.01, ***<0.001) (B) Sample distribution at third-day post symptoms using the cytokines showed in (A) based on principal component analysis.

Based on the significant severity-specific temporal protein dynamics, our analysis identified a signature of nine cytokines at 3 DPS whose dysregulation can be used as a predictive marker of severe Dengue (Fig 2A). The dysregulation of these signature cytokines was highly correlated with the severity of the DENV, as observed in the principle component analysis (Fig 2B). Notably, the levels of IL-6, IL-8, IL-10, IL-13, and GM-CSF were markedly upregulated in severe Dengue patients on day three after febrile symptoms compared to other groups while a decrease in MIP-1β and IL-12, suggesting that a panel of these cytokines might aid in the prediction of Dengue severity. Elevated levels of IL-6, IL-8, and GM-CSF can help activate and recruit immune cells, promote inflammation, and facilitate the clearance of DENV. While IL-10 may contribute to the suppression of the immune system, leading to an inadequate antiviral response and an uncontrolled inflammatory reaction, which in turn contributes to the development of severe dengue (Tsai et al., 2013). Recent studies and meta-analyses have identified some of these markers as early predictive biomarkers for severe Dengue progression (Flores-Mendoza et al., 2017; Moallemi et al., 2023; Soo et al., 2017).

Similarly, a strong correlation between GM-CSF and thrombocytopenia has been recognized as a hallmark of severe Dengue (Flores-Mendoza et al., 2017; Gowri Sankar and Alwin Prem Anand, 2021; Soo et al., 2017). IFN-γ is secreted by activated T and natural killer cells activated during Dengue infection. This, in turn, leads to the activation of macrophages and dendritic cells. However, even though levels of IFN-γ are similar in patients at various stages of Dengue infection, it is still unclear why the subsequent cytokine levels are higher in patients with severe Dengue than mild ones. Recently, it was observed that the synergistic action of TNFα and IFN-γ leads to inflammatory cell death, thereby initiating the cascade of the cytokine storm. The rapid cell death in severe Dengue might contribute to increased cytokine levels.

In conclusion, using longitudinal cytokine profiling, we have identified that the third-day post symptoms cytokine profile predicts severe dengue due to a disrupted balance of pro- and anti-inflammatory in secondary dengue infection. It may help in early clinical decision-making to treat severe dengue infection.

## CRediT authorship contribution statement

**Puneet Bhatt:** Writing – review & editing, Methodology, Investigation, Formal analysis, Data curation, Conceptualization. **Muralidhar Varma:** Supervision, Resources, Data curation, Conceptualization. **Vikas Sood:** Writing – review & editing, Validation, Investigation. **Anoop Ambikan:** Writing – review & editing, Visualization, Software, Methodology, Formal analysis. **Anup Jayaram:** Writing – review & editing, Formal analysis, Data curation. **Naren Babu:** Writing – review & editing, Formal analysis, Data curation. **Soham Gupta:** Writing – review & editing, Validation, Supervision, Formal analysis. **Chiranjay Mukhopadhyay:** Writing – review & editing, Supervision, Funding acquisition, Conceptualization. **Ujjwal Neogi:** Writing – original draft, Visualization, Supervision, Resources, Methodology, Conceptualization.

## Declaration of competing interest

The authors declare that they have no known competing financial interests or personal relationships that could have appeared to influence the work reported in this paper.

## Data Availability

Data will be made available on request.

## Appendix Supplementary materials

Image, application 1
