# Supplementary material for: Temporal cytokine storm dynamics in dengue infection predicts severity
Source: Virus Res. 2024 Jan 6;341:199306. doi: 10.1016/j.virusres.2023.199306 (PMC10818250; doi:10.1016/j.virusres.2023.199306)
Supplement: Supplementary file 1 [file mmc1.docx]

**Supplementary Table 1. Temporal cytokine profiling in DENV patients.**

| **N** | 17 | 59 | 50 | 44 | 26 | 13 | 42 | 25 |
| --- | --- | --- | --- | --- | --- | --- | --- | --- |
| **DPS** | **2** | **3** | **4** | **5** | **6** | **7** | 3 to 5 DPS§ | 3 to 6 DPS§ |
| **Cytokines** |  |  |  |  |  |  | *Adj p values* | *Adj p values* |
| IL-17 | 2.34 (2.01-3.66) | 4.12 (2.54-5.54) | 2.75 (2.05-4.1) | 1.75 (1.12-1.28) | 1.12 (1.12-1.3) | 1.12 (1.12-1.12) | <0.0001 | <0.0001 |
| IL-13 | 1.51(1.13-2.29) | 7.72(4.12-16.15) | 10.05(8.13-20.98) | 14.21(9.16-31.04) | 19.42(7.87-29.79) | 10.43(7.41-31.19) | <0.0001 | <0.0001 |
| TNF-α | 12,52(7,64-15,345) | 12,13(9,71-17,19) | 8,55(4,5-13,56) | 3,645(1,5-10,0475) | 1,5(1,5-3,41) | 1,5(1,5-1,5) | <0.0001 | <0.0001 |
| IL-12 | 0,845(0,7225-1,27) | 2,23(0,965-4,37) | 3,115(1,12-5,7075) | 1,17(0,845-2,2275) | 0,845(0,845-0,9925) | 0,845(0,845-0,845) | <0.0001 | <0.0001 |
| IL-6 | 1,59(0,215-15,19) | 3,2(0,215-19,59) | 1,03(0,215-7,7825) | 0,935(0,215-3,195) | 0,215(0,215-0,23375) | 0,215(0,215-0,61625) | <0.0001 | <0.0001 |
| IFNγ | 3,43(0,645-6,98) | 2,98(1,555-4,87) | 2,24(0,58-3,93) | 1,19(0,26-3,545) | 1,16(0,26-2,06) | 0,45(0,26-2,4275) | <0.0001 | 0.0293 |
| IL-7 | 2,76(1,4-6,955) | 4,72(1,79-9,08) | 3,855(1,4-8,5025) | 3,14(1,4-6,955) | 3,14(1,4-6,7075) | 1,4(1,4-6,67) | 0.0169 | 0.0609 |
| IL-4 | 0,26(0,08875-0,5975) | 0,26(0,085-0,87) | 0,45(0,085-1,22) | 0,375(0,085-0,8525) | 0,2(0,085-0,725) | 0,1025(0,085-0,6225) | 0.0291 | 0.0346 |
| MCP-1 | 73,55(14,145-222,545) | 53,64(23,885-128,35) | 44,42(25,06-69,785) | 39,28(21,615-71,535) | 27,535(16,22-64,4475) | 30,91(25,3975-50,6775) | 0.0396 | 0.0611 |
| MIP-1β | 35,67(22,38-115,53) | 46,47(26,88-77,71) | 49,21(37,025-97,28) | 37,285(22,705-58,3075) | 31,345(19,21-42,285) | 25,79(15,28-51,2025) | 0.0551 | 0.0079 |
| IL-8 | 96,04(22,245-170,65) | 52,61(17,155-135,23) | 44,565(16,1375-202,035) | 34,36(16,8975-142,73) | 18,57(8,4675-120,22) | 22,08(8,63-45,99) | 0.0590 | 0.0307 |
| IL-10 | 12,11(4,485-35,82) | 16,19(6,555-61,56) | 17,105(6,8425-55,7575) | 20,45(2,8025-112,3725) | 8,84(2,4825-22,895) | 5,375(3,135-16,05) | 0.1647 | 0.0031 |
| G-CSF | 3(2,025-7,665) | 3,24(2,025-9,22) | 2,1025(2,025-8,195) | 3,35(2,025-8) | 2,025(2,025-5,215) | 3,44(2,025-7,205) | 0.3541 | 0.2968 |
| IL-1β | 0,995(0,7625-6,79) | 0,55(0,305-1,75) | 0,535(0,355-1,3625) | 0,46(0,1575-1,4225) | 0,42(0,1325-2,505) | 0,52(0,13-1,0975) | 0.5855 | 0.6791 |

Footnote: IL-2, IL-5, and GM-CSF were mainly detected in severe dengue cases. §Samples available for all the time points
